# A method for analyzing text using VOSviewer

**DOI:** 10.1016/j.mex.2023.102339

**Published:** 2023-08-22

**Authors:** Umar Ali Bukar, Md Shohel Sayeed, Siti Fatimah Abdul Razak, Sumendra Yogarayan, Oluwatosin Ahmed Amodu, Raja Azlina Raja Mahmood

**Affiliations:** a Centre for Intelligent Cloud Computing (CICC), Faculty of Information Science and Technology, Multimedia University, Melaka, Malaysia; b Department of Electrical, Electronics Systems Engineering, Universiti Kebangsaan Malaysia (UKM), Bangi, Selangor 43600, Malaysia; c Information and Communication Engineering Department, Elizade University, Ilara-Mokin, Ondo State, Nigeria; d Department of Communication Technology and Network, Faculty of Computer Science and Information Technology, Universiti Putra Malaysia, Serdang, Selangor Darul Ehsan 43400, Malaysia

**Keywords:** Dataset, Text analysis, Visualization, VOSviewer, Analysis and visualization of text data

## Abstract

The need for technical support for data handling and visualization solutions has increased in tandem with the complexity of today's data and information, that is of multiple sources, huge in size and of different formats. This study focuses on handling and analyzing text-based data. Despite many available text analysis tools, there is a high demand among researchers for easy- to-use tools yet scalable and with incomparable visualization features. Of recent, there has been a significant focus on utilizing VOSviewer, an open-source software for bibliometric analysis. This software is able to analyze a significant amount of data and provide excellent network data mapping. However, there is a lack of existing work in evaluating this sophisticated tool for text analysis. Thus, this article explores the capability of VOSviewer and presents evidence-based implementation of this software for text analysis.

Specifically, this study demonstrates the usage of VOSviewer to analyze text based on YouTube interviews related to ChatGPT. Hence, this study significantly contributes by processing textual data and producing visualization network maps that are different from bibliometric data. The study recognizes VOSviewer as a powerful tool for data visualization in mapping text data and illustrates the potential of this software for analyzing text networks in various fields.

• The study illustrates how text analysis and visualization can be realized using VOSviewer, an open-source software mostly used for biblio- metric analysis.
• The study presents the workflow indicating how the dataset can be prepared as input for VOSviewer for text analysis.
• The study proves that VOSviewer is a powerful tool for data visualization and network mapping for any type of network data including transcripts from social media.

## Graphical abstract

**Figure:**
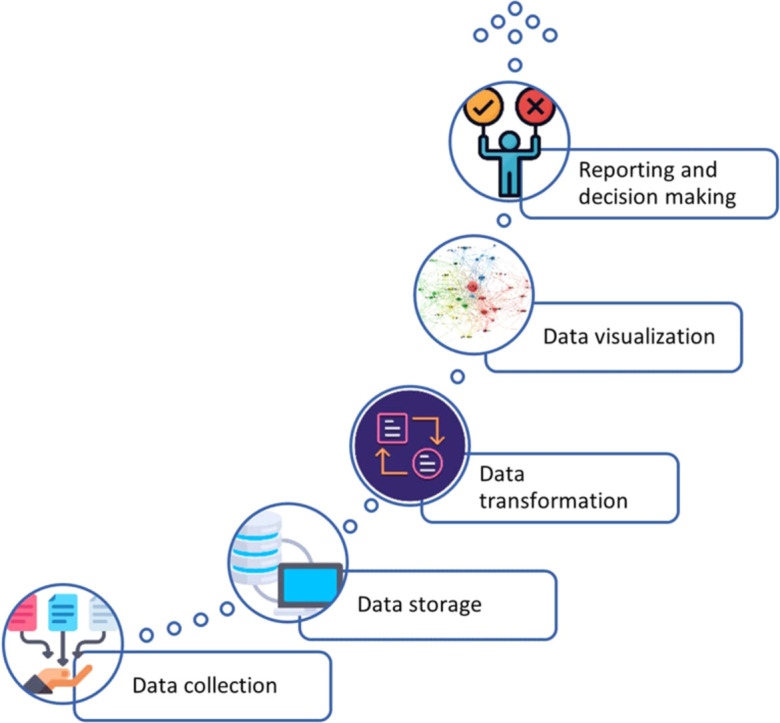

Specifications table

**Table:** 

Subject area:	General disciplines
Specific subject area:	Data science- text analysis
Method name:	Analysis and visualization of text data
Name and reference of original method:	VOSviewer
Resource availability:	Microsoft Excel, Lexos, and VOSviewer

## Introduction

The advent of smart technologies and the emergence of ‘big data’ has led to an increased demand for data analysis and management techniques in several disciplines. Particularly, the ever-increasing volume and variety of data necessi- tate the deployment of easy-to-use solutions to analyze data comprehensively. This requires the integration of efficient tools that cater to the technical demands of several disciplines and requirements for various stages of data processing such as the gathering of data, cleaning of data, analysis, and visualization [12]. These increases in data processing demands constitute a challenge [16], especially since extrapolating patterns and visualizing large data sets is becoming more difficult [3,8,12,13] due to the ever-increasing complexity of generated data. Thus, visualization tools to draw inferences from such data are highly sought after in view of the need for quality decision-making.

There are many text analysis solutions currently available, yet there is still a need for sophisticated yet easy-to-use tools for text analysis with graphical representation capability. The text analysis and production of visualization networks generally require some technical expertise and some knowledge of data analytics. This could be a time consuming, expensive, and laborious process. Moreover, conventional solutions may require knowledge of certain programming languages, and this could be difficult due to the complexity of the data. Researchers or even data scientists experience difficulties, especially due to time constraints in handling and processing a large amount of data to provide significant insights into the data in a timely manner. Therefore, it is necessary to develop advanced methods of visualizing complex information for effective interpretation of the data, which should be able to process a significant volume of data in a short time and provide significant insights based on the generated visualization maps.

VOS ("visualization of similarities") was a concept developed less than two decades ago for analyzing and visu-alizing patterns within data [4]. Years later, the VOS concept was developed into a program called VOSviewer for bibliometric analysis, hence has been widely adopted in bibliometric and citation studies for constructing and visualizing bibliometric networks, with journals, researchers, or individual publications as actors, based on co-citation, bibliographic coupling, or co-authorship relations Van Eck and Waltman [21]; Moral-Muñoz, Herrera-Viedma, Santisteban-Espejo and Cobo [14]. The VOS offers the possibility of building co-occurrence networks of important terms extracted from a corpus of scientific literature using text-mining functionality. VOSviewer was developed by the center for Science and Technology Studies (CWTS) at Leiden University in The Netherlands. It can extract bibliographic networks from bibliographic data based on data files downloaded from WoS, Scopus, Dimension, PubMed, and RIS format. VOSviewer is a Java-based application that can be used to generate maps based on network data, as well as visualize and examine these maps. However, despite its potential, there is a limited number of studies that utilized the VOSviewer for text analysis and visualizations in a setting other than that involving a bibliometric dataset. While there are numerous programs and tools for bibliometric analysis such as CiteSpace, HistCite, and CitNetExplorer, VOSviewer boasts excellent visualization capabilities and can efficiently handle data import and export from various sources [14].

Hence, this paper provides a method for utilizing VOSviewer to manage text and produce a visualization map of the dataset. Particularly, it demonstrated how VOSviewer can be used to process, store, and analyze data collected from social media including transcripts obtained from YouTube. The significance and potential use case of the study lie in addressing the increasing demand for easy-to-use yet scalable tools for handling and analyzing complex text- based data. The study highlights the untapped potential of utilizing VOSviewer, originally developed for bibliometric analysis, as a powerful tool for text analysis and data visualization. In particular, the study discusses the steps required for data processing; data collection, and manipulation using ChatGPT-related YouTube interviews as a case study, and presents the methodology to perform the analysis for reporting purposes and decision-making. Particularly, emphasis is made on how VOSviewer can be used to process, store, and analyze unstructured social media data. The methods and techniques utilized in processing the raw data until the production of the visualization map are detailed. Therefore, the fundamental objectives of this paper are detailed below:

• To define the techniques and methodologies used for processing the raw textual data video data for VOSviewer software to generate network maps.
• To develop an information workflow that is tailored to the specific needs of text analysis which is different from bibliometric analysis that can be applied to a variety of disciplines and topics.
• To demonstrate the applicability of VOSviewer and produce the co-occurrence networks of video data different from bibliographic data.

Therefore, the remaining part of this paper is organized as follows: A description of the method, which covers the workflow of the data processing and analysis, dataset structure, as well as VOSviewer description, deployment, and operation are presented. Finally, the conclusion, limitations, and implications are provided in concluding remarks section.

## Method details

**Fig. 1:**
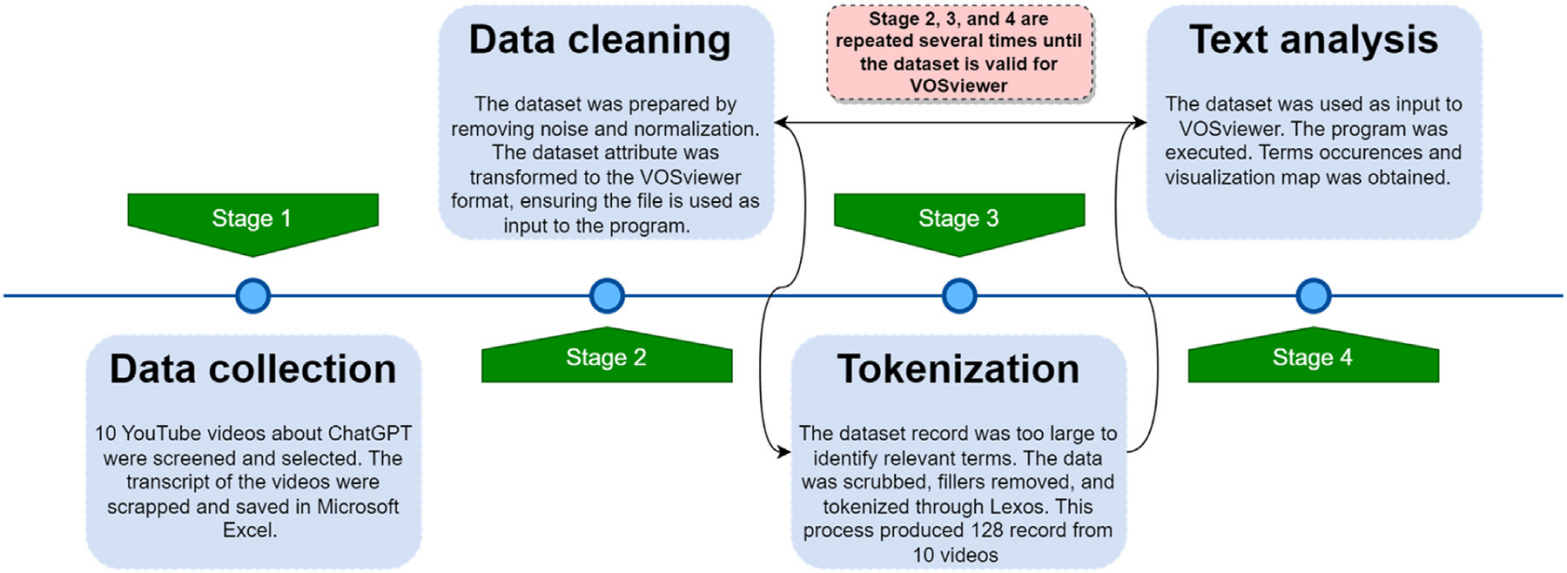
This is the workflow overview that was developed in order to visualize the data from YouTube. The directions of the workflow are indicated by stage 1 to stage 4; starting from left to right. The data were manually collected from YouTube by the researchers (UA Bukar and MD S. Sayeed) and stored in Microsoft Excel spreadsheets. Next, the dataset attribute was transformed according to the VOSviewer's format due to the large text found in the record. Lexos was used to scrub noise and fillers and perform tokenization of the data. In order to visualize the data in an interactive manner, the dataset file was used as an input to VOSviewer.

**Fig. 2:**
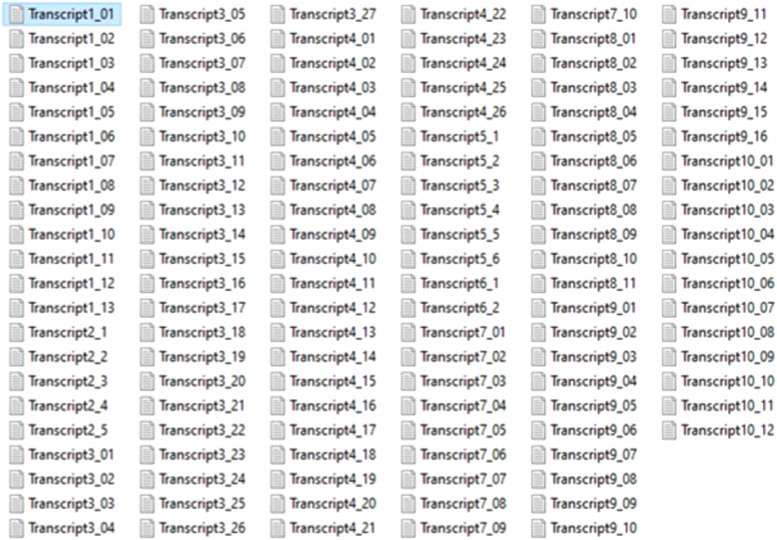
Tokenized transcripts of the expert interviews.

**Fig. 3:**
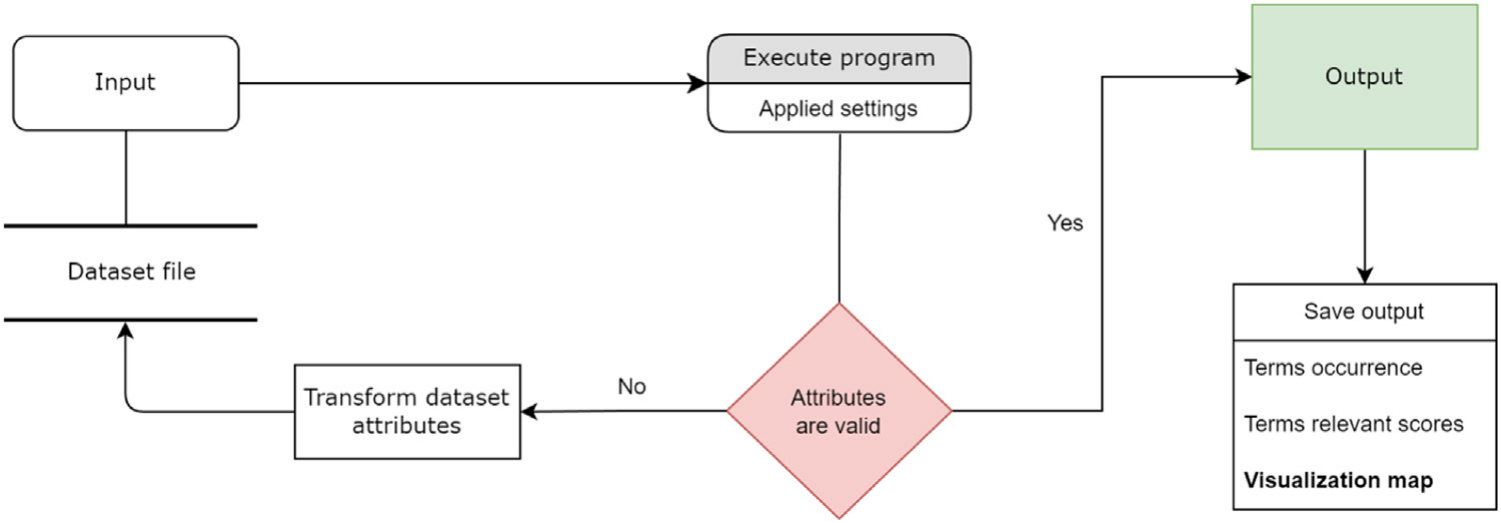
The execution of VOSviewer: a simplified directed process of the execution of VOSviewer. The initial data and final dataset are hosted on Mendeley data (The VideoTranscript and Dataset of Expert Opinion of ChatGPT).

The transcript of the data (YouTube videos on ChatGPT) was collected. Note that the accuracy and comprehensiveness of the results are limited to the coverage and quality of the underlying database, which may affect the analysis and visualization generated by VOSviewer. Considering the fact that this study extracted the data from a YouTube video which is not a default bibliometric data. The process of cleaning and transforming the data to meet up with VOSviewer requirements is one of the key strengths of the paper. The following sections demonstrate how the dataset can be modified to perform other analyses different from bibliometrics (Refer to Figs. 1, Fig. 2, Fig. 3, and Table 1).

**Table 1:** ChatPT dataset heading format for VOSviewer analysis.

S/N	Video title	Source title	Facilitators/ host	Expert name	Expertise	Video link	Transcript	Initial Attributes
S/N	Title	Source title	Facilitators/ host	Authors	Author Keywords	Video link	Abstract	**Attributes used for VOSviewer**

### Workflow description

This study designed a workflow for the purpose of manipulating and visualizing data for text analysis of the ChatGPT YouTube video interviews. The proposed method of text analysis is broken down into four primary stages. Fig. 1 depicts the architecture of the proposed method in this study. The diagrammatic representation of the overall process flow concentrates on the management of data. Hence, the workflow includes processing the data from the data sources to the visualization solution. The key stages are described in the proceeding sections.

• **Stage 1: Data collection**. This involves acquiring data from YouTube about ChatGPT. Ten videos were selected through screening, ensuring that the videos were expert interviews about ChatGPT. The transcripts of the videos were saved in Microsoft Excel.
• **Stage 2: Data cleaning**. Data cleaning is often needed for the creation of network maps based on text data [22], which is essential for computational-based text analysis to generate meaningful insights. First, the data was pre-processed in Microsoft Excel. This was carried out for the purpose of filtering out irrelevant information (i.e., punctuation, noise removal, and symbols) from the data. At this stage, VOSviewer only accepts bibliometric datasets as input. As a result, the attributes of the dataset about ChatGPT were formated in accordance with the VOSviewer's requirement.
• **Stage 3: Tokenization**. Lexos software was utilized for additional data cleansing and transformation through scrubbing and tokenization. The Lexos, an integrated lexomics workflow, is a web-based tool that assists users in exploring a preferred collection of digitized texts through computational and statistical techniques on the corpus of texts, which ensures a more thoughtful and informed approach to text analysis, empowering researchers to uncover valuable insights from their corpus of texts [11]. Data scrubbing is the process of removing personally identifiable information from the text [1], whereas tokenization is the process of dividing the lengthy text into segments or tokens for text analysis [25]. The study observed that the record of the dataset is too large to be used in map generation. Lexos was then adopted to further scrub the data and tokenize them into chunks, thus improving the VOSviewer text analysis process.
• **Stage 4: Text analysis**. The tokenized dataset is fed into the VOSviewer. The software identifies the most frequent terms used by the experts and the resulting visualizations show clustering of relevant terms, known as co-occurrence maps. VOSviewer was adopted as it has been considered an outstanding text mapping tool for scientific mapping [4,5,7,26] and this work, has been extended to generate maps based on tokenized text data. Although VOSviewer was primarily designed for evaluating bibliometric networks, it can be used to create, analyze, and investigate maps based on any type of network data [7]. In summary, the dataset was provided as an input to VOSviewer, which was then processed and finally, visualization maps were generated based on the textual data. In addition, it computed the relevance scores between these various terms and using these networks, the relationships between the experts’ terms were determined.

**Fig. 4:**
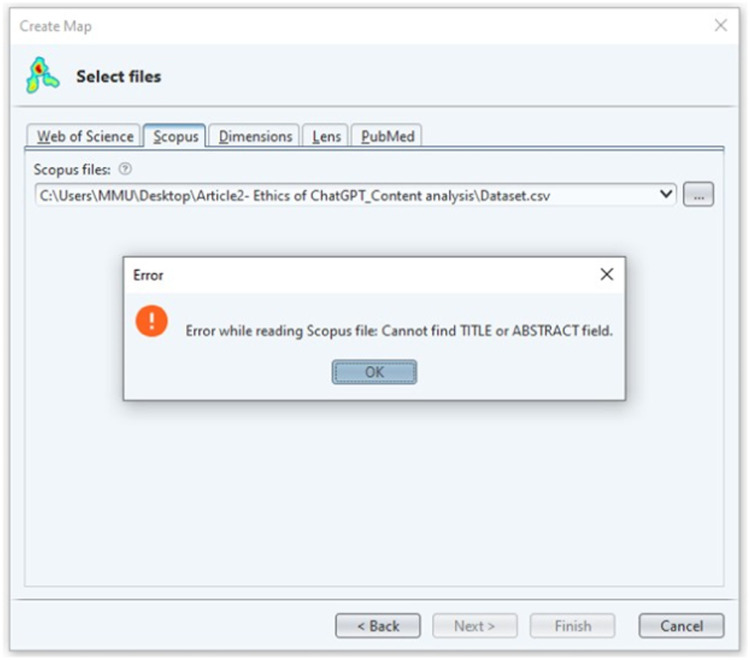
Error message for invalid data input: *Note*: The error message occurs if the column field is not formated according to the VOSviewer format.

The tokenization procedure generated 128 text files, which were uploaded into Microsoft Excel in the form of records from the 10 videos’ content, as shown in Fig. 2. Additionally, Fig. 3 illustrates a simplified data flow diagram of the execution of the VOSviewer for text analysis, which is different from bibliometric analysis. Based on the VOSviewer settings, Scopus is the common database from which the data files are retrieved. Hence, it is necessary to transform the dataset according to the format used in Scopus files, as it is being considered in this study. Firstly, the dataset file was uploaded as an input to the VOSviewer program. This process started when the VOSviewer detected the file as a valid file. If the file dataset is not valid, an error message will appear indicating the file cannot be executed. Fig. 4 shows the respective error message display. This specific error message indicates that some attributes of the dataset must have TITLE and ABSTRACTS labels in the Excel input file. The process of transforming the input data file to meet the software's requirement was repeated several times until no errors were found and the file was ready for further analysis.

## Dataset structure

**Fig. 5:**
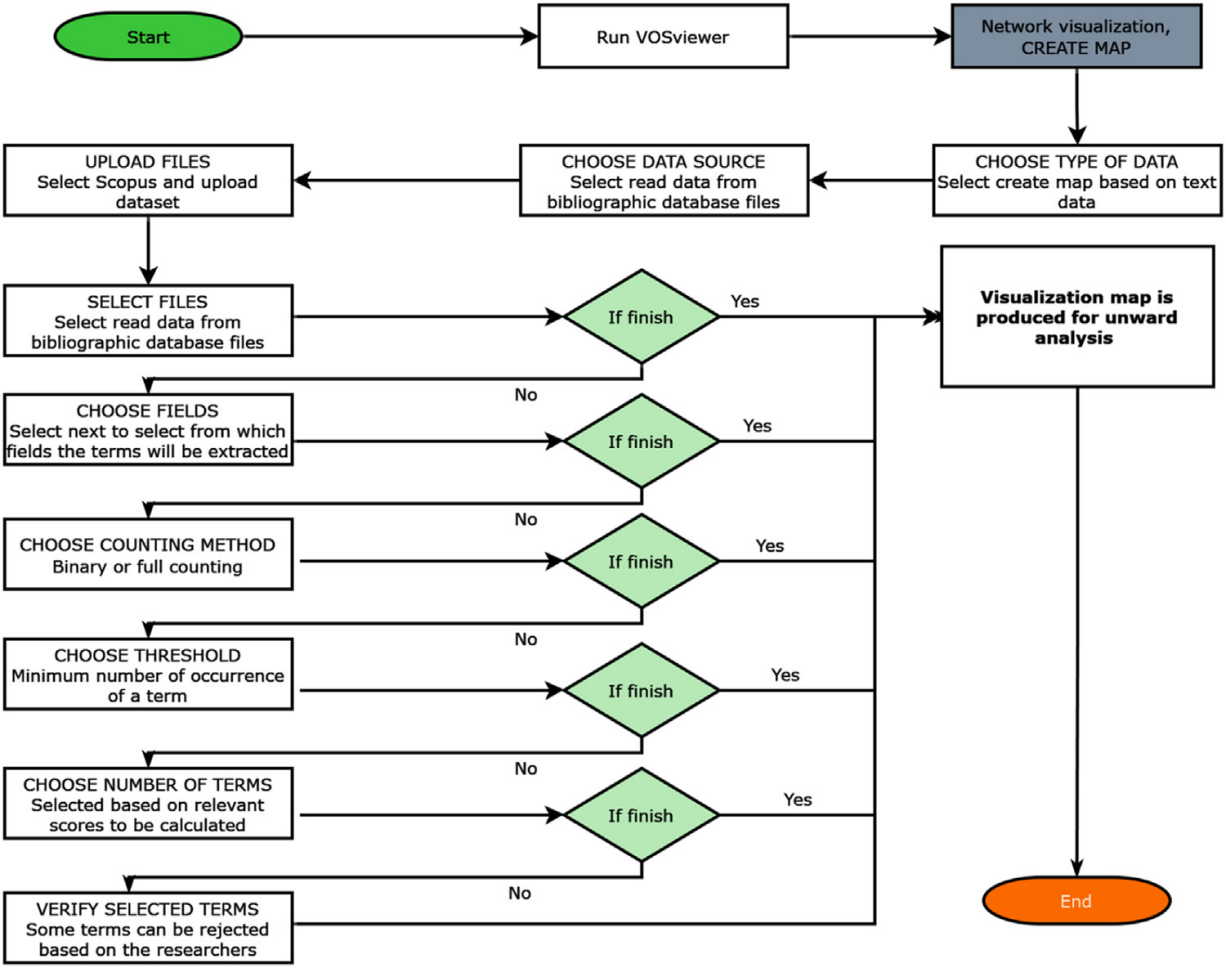
Seamless execution of VOSviewer for text analysis with valid dataset.

Many researchers analyzing bibliometric networks [6,17,20,27] have validated the effectiveness of VOSviewer in their studies. The efficacy of the VOSviewer in visualizing and generating text maps based on bibliometric data is outstanding [26]. However, VOSviewer cannot work without specifying fields for Author, Abstract, Keyword, etc. in the Excel input file. Thus, the column of the text to be analyzed must be read as ``Abstract'', and the identification column must be read as ``Author''. The structure of the data heading used in the text analysis is presented in Table 1. A detailed flowchart demonstrating the application of text analysis on VOSviewer is presented in Fig. 5. The graphical representation of the various stages of VOSviewer implementation is presented in Appendix A (refer to Fig. 7). Seamless execution can only be obtained if a valid dataset and data structure is fed into the program.

### VOSViewer Deployment and operation

A database file must be submitted as input or retrieved via an embedded application programming interface (API) in order to construct a network. The VOSviewer program analyzes the input file, generates the network map and then provides map visualization and map exploration functions. The VOSviewer provides access to three distinct maps: network visualization, overlay visualization, and density visualization. The network visualization and density visualization features of VOSviewer make it an exceptional tool for scientific knowledge mapping [26]. Using these networks for diverse objects based on modified metrics (such as users’ remarks, keywords, geographical and affiliations, etc.), the relationships and correlations between items can be displayed in an understandable manner. Consequently, this study utilized these capabilities to generate, analyze, and investigate maps based on the ChatGPT dataset.

The generated visualization map displays the keywords or terms in the data file based on the clustering techniques available in VOSViewer. The approach is based on mapping and clustering derived from the same underlying princi- ple [24]. This was established based on the VOS mapping technique, which was proposed as an alternative to the well-known multidimensional scaling (MDS) technique [4,23] and the clustering method based on a weighted and parameter- ized variant modularity function [15]. Hence, refer to Waltman et al. [24] for a detailed discussion of the mathematical modeling, demonstrated in Equations 1 to 7. The unified VOSviewer clustering technique can be seen as a kind of weighted variant of modularity-based clustering which has a resolution parameter to identify small clusters. Moreover, the command line parameters related to clustering techniques in the VOSViewer program can be found in Van Eck and Waltman [22] and Eck and Waltman [7].

**Fig. 6:**
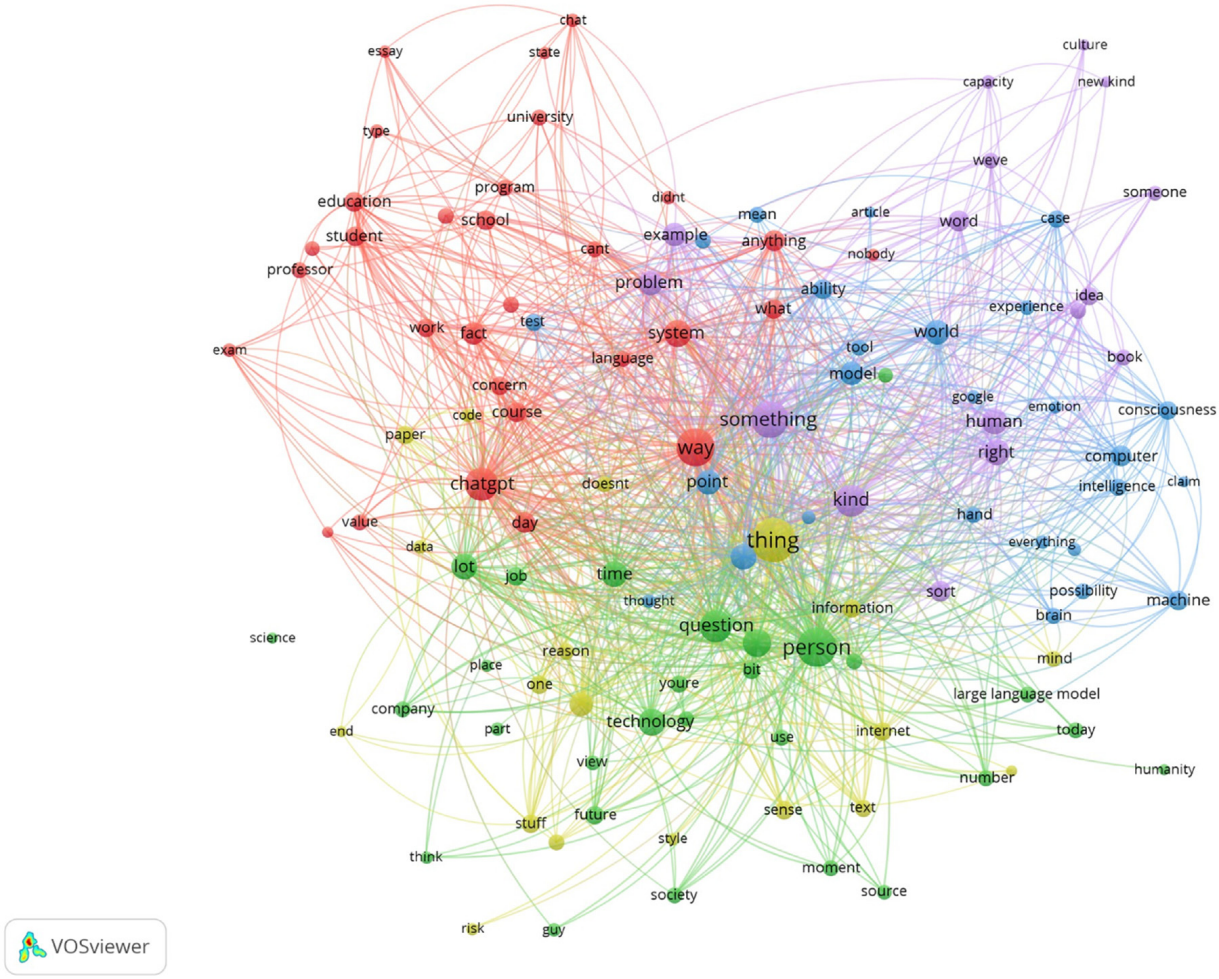
Visualization network map from the expert videos transcript.

**Fig. 7:**
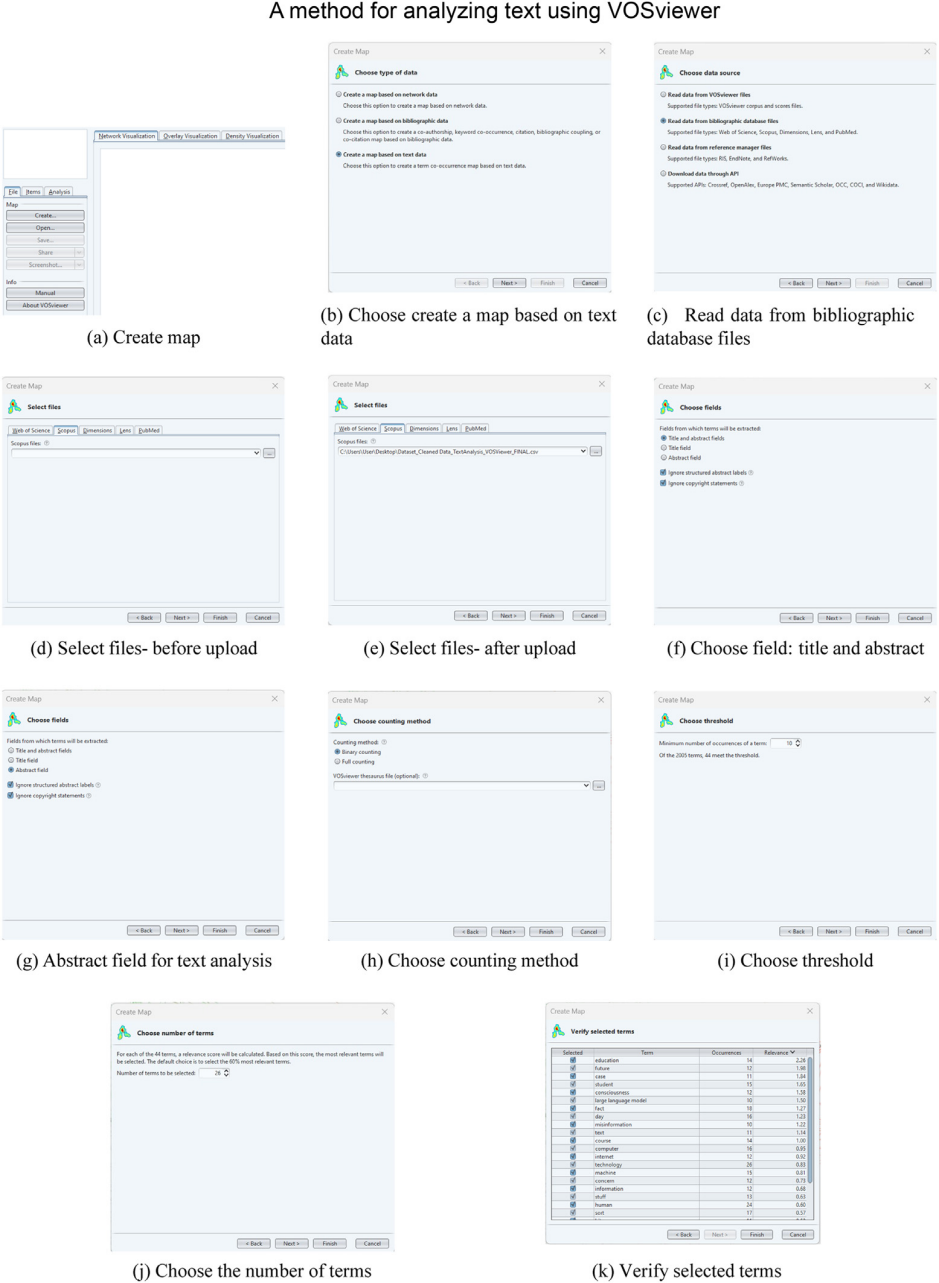
Screenshot showing seamless execution of VOSviewer for text analysis.

Accordingly, the visualization map of term occurrence depicts the frequency of occurrence of certain key terms, hence called occurrence metric. The terms are represented as nodes of varying sizes, proportional to the terms’ recorded frequency. Additionally, the analysis indicates the frequency with which the terms appear in close proximity to one another. The co-occurrence of terms within a text network has a substantial effect on the construction of text clusters, also known as communities of terms. The term ``community of terms'' refers to a group of terms that cluster collectively in the text network. Multiple instances of communities can exist on a text network (see Fig. 6). The VOSviewer's textual clustering method was used to determine the primary cluster prevalent for each text. The purpose of this strategy is to produce a number of clusters, with each cluster representing a distinct ChatGPT-related topic. For example, in Fig. 6, five clusters were created within this demonstration; red, green, purple, blue, and yellow. It is worth noting that a few generic and insignificant terms such as doesn't, you're, didn't, guy etc. were also listed in Fig. 6 for having high occurrence values. Therefore, to ensure insightful and accurate analysis can be performed on the text of interest, further data processing such as removing the generic and irrelevant terms is required (refer to Bukar, Sayeed, Mahmood, Abdul Razak, Yogarayan and Amodu [2]), which is beyond the discussion of this paper.

Moreover, the co-occurrence analysis, as employed in VOSviewer, focuses on identifying relationships based on the frequency of appearance of items within the same context, without explicitly considering the strength or directionality of connections. However, we would like to clarify that our method leverages VOSviewer's co-occurrence maps as an initial step in the analysis process rather than the sole means of interpretation. The co-occurrence maps serve as a valuable starting point to identify prominent relationships between items, such as keywords. These visualizations offer a high-level overview of the interconnectedness among items, helping this study to identify relevant themes and clusters within the dataset. Nevertheless, this study recognizes the importance of delving deeper into the data to understand the strength and directionality of relationships, as well as potential nuances that co-occurrence analysis may overlook. Refer to Bukar et al. [2] which provides an in-depth analysis of the textual content of the items. This covers the insights that are derived from the analysis which is able to capture the semantic meaning or context of the items in the visualization networks.

## Concluding remark

This study was performed to present an easy-to-use and effective solution to analyze textual data with excellent visualization features. VOSviewer has been proven efficient for bibliometric analysis, and hence this study adopted this software to perform text analysis on expert videos and interview data about ChatGPT that were downloaded from YouTube. The processes involved in handling, preparing and analyzing the textual data have been presented and discussed. This includes the detailed workflow representing the stages involved in preparing and analyzing the data as well as discussion on the resulting graphs. Thus, this study shows that there is a potential for using VOSviewer for text analysis as the resulting visual networks, via the co-occurrence maps that are able to provide meaningful data insights.

The significance of the study lies in its ability to address the demand for efficient text analysis tools, explore VOSviewer's adaptability for text-based data, and present evidence-based implementation of the software. The po- tential use cases include facilitating text data analysis, visualizing complex textual information, and inspiring diverse applications in various research domains beyond bibliometrics. Specifically, the study specifically focuses on analyz- ing text-based data from YouTube interviews related to ChatGPT. However, the potential use cases of VOSviewer for text analysis extend beyond this specific context. Researchers and professionals from various fields can draw inspi- ration from this work and explore VOSviewer's application for analyzing text networks in their respective domains. Additionally, by evaluating and showcasing the application of VOSviewer for text analysis, the study adds to the reper- toire of research methodology options available to scholars. This contribution fosters innovation in data handling and visualization techniques, which can benefit future studies across different disciplines.

Although the study observed some limitations, for example, the lack of stemming functionality of VOSViewer, which means it doesn't generate morphological variations of root words [9,10]. Additionally, VOSviewer lacks the capability for temporal analysis, a feature readily available in tools like R such as Xplortext with a set of functions for multivariate exploratory analysis on textual data [18]. Moreover, VOSviewer's visualizations are mostly limited to network graphs, and it does not support other types of visual representations like geospatial maps, tree maps, spectrograms [19], or data different from the bibliography dataset. When a map is created based on text data, there often is a need to perform data cleansing [22]. This led the researchers in this study to combine VOSviewer with Lexos. Using a mix of tools to break the weakness of VOSViewer beyond bibliometric analysis. Hence, to enhance the data analysis further, the study proposed the development and integration of additional tools that offer thematic analysis options. This would involve plotting clusters of keywords along two dimensions (density vs. centrality) [19], enabling the identification of emerging clusters and determining their level of significance. This feature proves to be highly valuable for understanding the importance of each cluster. Assuredly, such improvements would have made VOSviewer an appealing data analysis tool for researchers across disciplines.

## CRediT authorship contribution statement

**Umar Ali Bukar:** Conceptualization, Data curation, Investigation, Methodology, Formal analysis, Visualization, Resources, Writing – original draft, Writing – review & editing. **Md Shohel Sayeed:** Funding acquisition, Project administration, Data curation, Resources, Writing – review & editing. **Siti Fatimah Abdul Razak:** Data curation, Project administration, Resources, Writing – review & editing. **Sumendra Yogarayan:** . **Oluwatosin Ahmed Amodu:** Conceptualization, Methodology, Formal analysis, Writing – review & editing. **Raja Azlina Raja Mahmood:** Conceptualization, Data curation, Writing – review & editing.

## Declaration of Competing Interest

The authors declare that they have no known competing financial interests or personal relationships that could have appeared to influence the work reported in this paper.

## Data Availability

The data is available at Mendeley Data: The Video Transcript and Dataset of Expert Opinion of ChatGPT.

## Appendix

A. Execution of VOSviewer with Valid Dataset

Graphical illustrations of the seamless execution of VOSviewer for text analysis are presented in Fig. 7.

## Appendix B Supplementary materials

Image, application 1
