# Supplementary material for: A method for analyzing text using VOSviewer
Source: MethodsX. 2023 Aug 22;11:102339. doi: 10.1016/j.mex.2023.102339 (PMC10491643; doi:10.1016/j.mex.2023.102339)
Supplement: Supplementary file 1 [file mmc1.docx]

**SUPPLEMENTARY FILE FOR PAPER ENTITLED “A METHOD FOR ANALYZING TEXT USING VOSviewer”**

**HOW TO RUN VOSviewer for TEXT ANALYSIS:**

The term "ChatGPT" was utilized as the search keyword to find ten (10) videos for analysis. These videos underwent a thorough assessment to determine if they were informative or served different purposes, and whether they provided information specifically related to the usage and applications of ChatGPT. After selecting the videos, their content was subjected to analysis using two tools: Lexos and VOSviewer.

Lexos was employed for data scrubbing and tokenization. Data scrubbing involves removing personally identifying information from the text to protect privacy, while tokenization divides the lengthy text into smaller chunks or tokens for effective text analysis. This process is crucial for extracting meaningful insights through computational-based text analysis. By using Lexos, 128 records were generated from the content of the 10 videos, providing a comprehensive dataset for further analysis.

Next, VOSviewer was adopted to create scientific maps based on the tokenized text data (**See Figure a to k**). Although VOSviewer was originally developed for evaluating bibliometric networks, it is versatile enough to construct, analyze, and explore maps based on any type of network data. The visualization process in VOSviewer utilizes a distance-based approach, where nodes are represented in a two-dimensional space. Nodes that are strongly related are positioned close to each other, while nodes with weaker relationships are located farther apart. The term "VOS" stands for "visualization of similarities," indicating its ability to showcase the connections between nodes based on their similarity.

In this study, the clean dataset resulting from Lexos' data scrubbing and tokenization was inputted into VOSviewer. The tool then processed the textual data and generated visualization maps that provided valuable insights into the relationships and patterns within the content of the 10 videos related to ChatGPT. This combination of analytical tools allowed for a thorough and detailed exploration of the videos' content and its relevance to the usage and applications of ChatGPT.

**
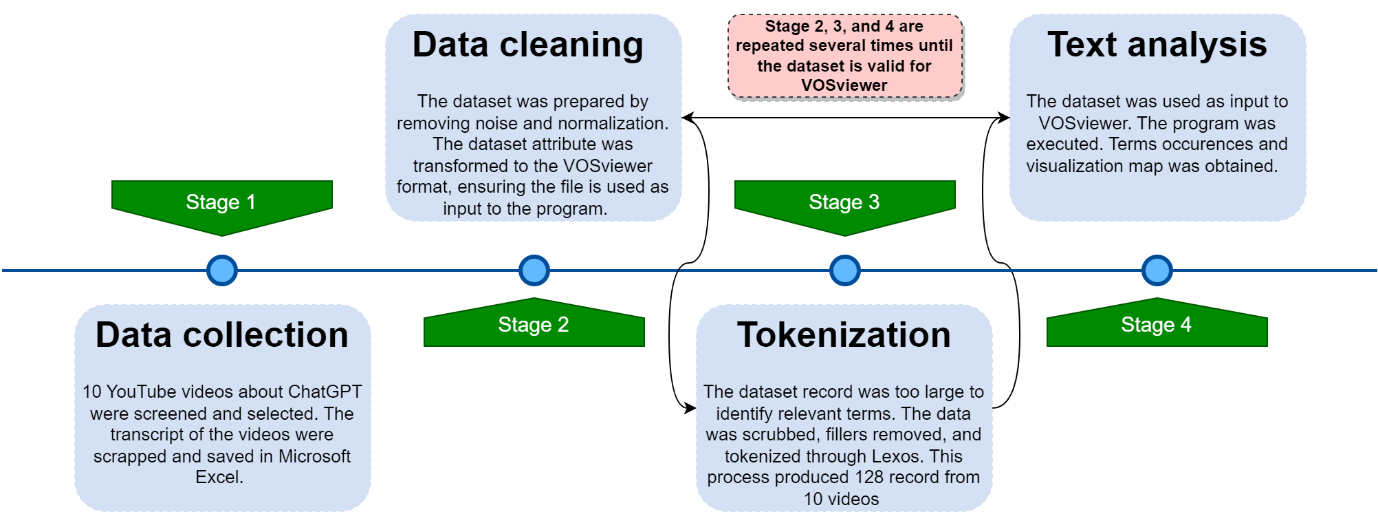
**

**Figure 1:** This is the workflow overview that was developed in order to visualize the data from YouTube. The directions of the workflow are indicated by stage 1 to stage 4; starting from left to right. The data were manually collected from YouTube by the researchers (UA Bukar and MD S. Sayeed) and stored in Microsoft Excel spreadsheets. Next, the dataset attribute was transformed according to the VOSviewer’s format due to the large text found in the record. Lexos was used to scrub noise and fillers, and perform tokenization of the data. In order to visualize the data in an interactive manner, the dataset file was used as an input to VOSviewer.

**Note:** The following screenshot (Figure a to k) demonstrate the seamless execution of VOSviewer for text analysis, with valid data file.


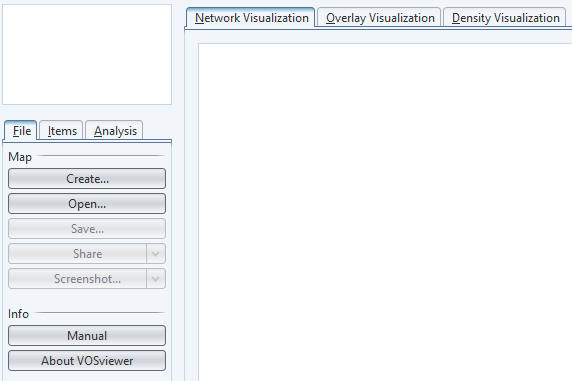


1. Create map: This is the first step for creating network visualization. The VOSviewer offers three types of visualization network, including network visualization, overlay visualization, and density visualization. From the right side, File is selected to create a map.


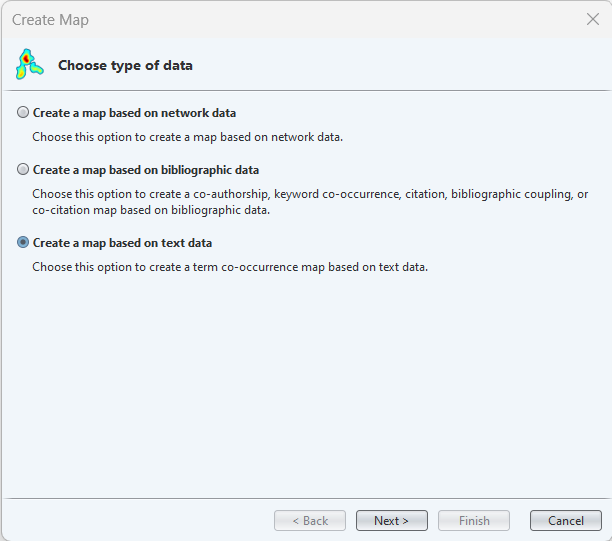


1. Choose create a map based on text data: Once create map is selected, the user choose create a map based on text data. This option is selected because the data is formatted to analyses text, instead of bibliometric such citation, co-authorship etc.


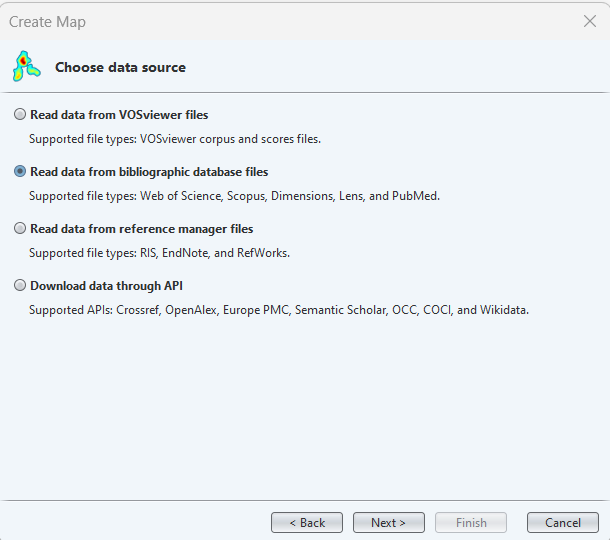


1. Read data from bibliographic database files: This option is selected to upload the data to create visualization network.


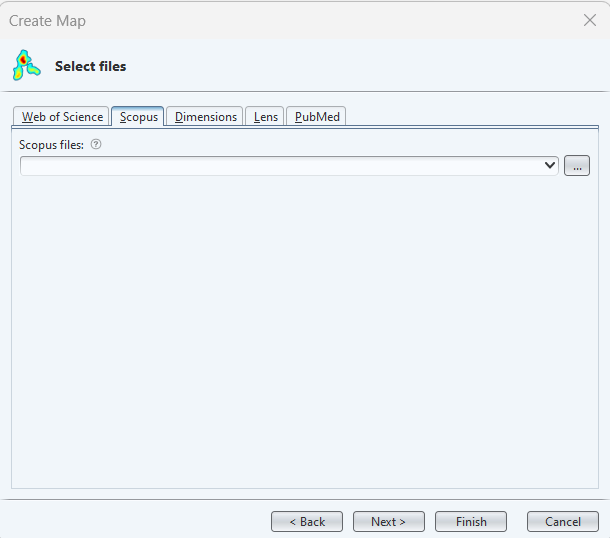


1. Select files- before upload: The is selected from the source.


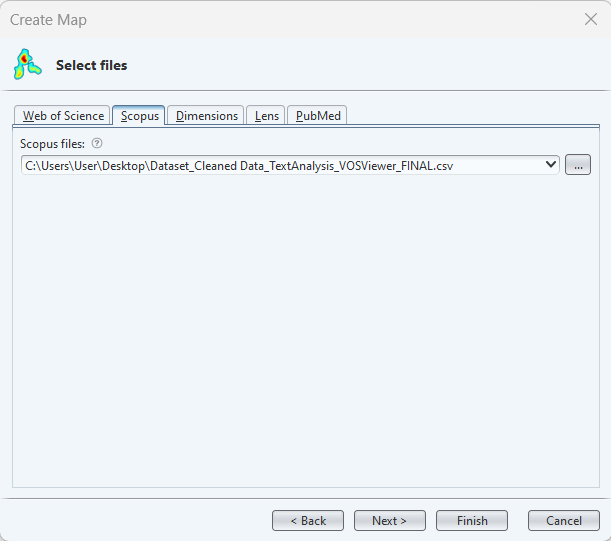


1. Select files- after upload: The file is uploaded, which is according to the format of VOSviewer.


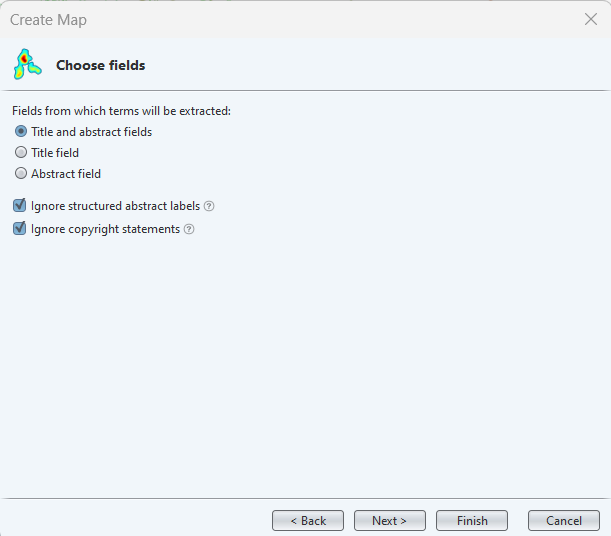


1. Choose field: title and abstract: When you doing text analysis, title and abstract fields is selected.


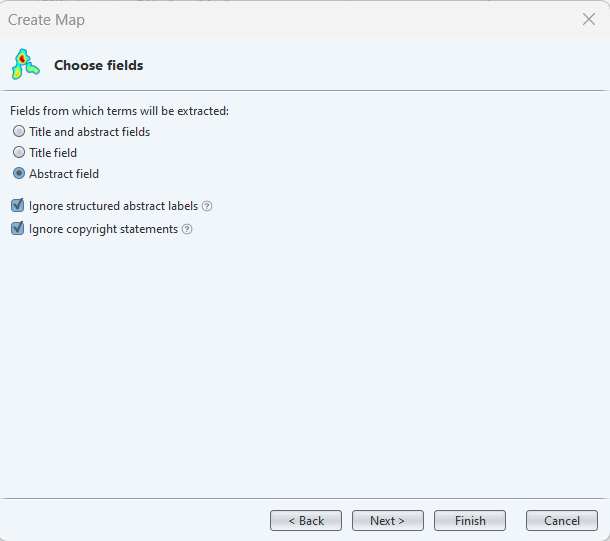


1. Abstract field for text analysis: Abstracts field is selected since the column field from the database file is rename as Abstract to enable VOSviewer accept the input files.


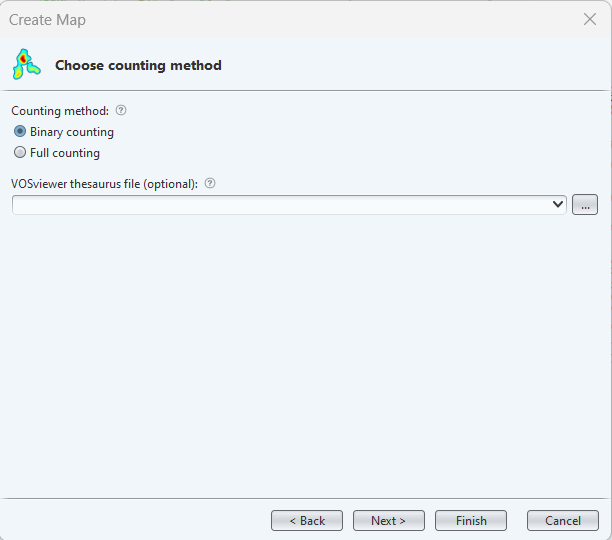


1. Choose counting method: The VOSviewer offers two counting method, any method can be selected according to the objective of the study.


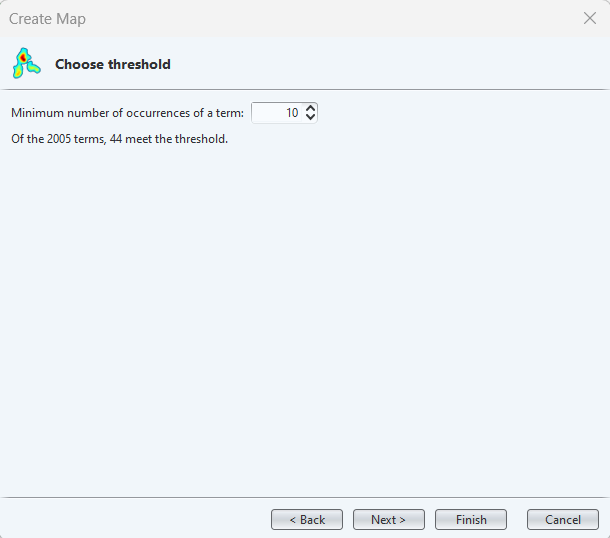


1. Choose threshold: The threshold specifies the minimum number of occurrences of a term. The more the number is selected, the more the terms to be considered in the visualization network. According this analysis, the total terms is 2005, and 44 meet the threshold based on 10 occurences.


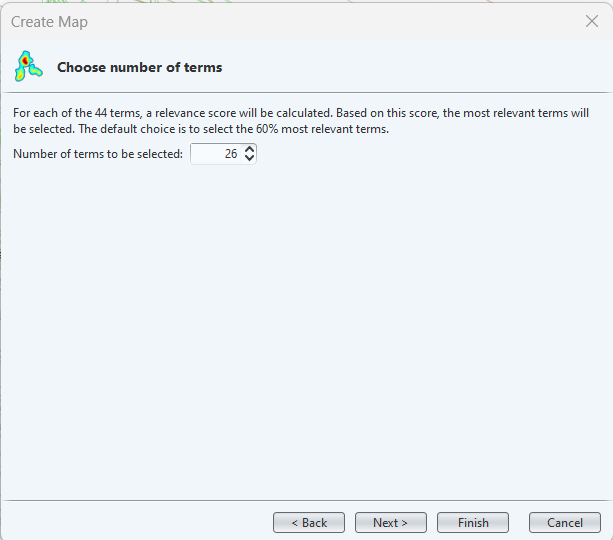


1. Choose the number of terms: This stage selects the numbers of terms based on the relevance score.


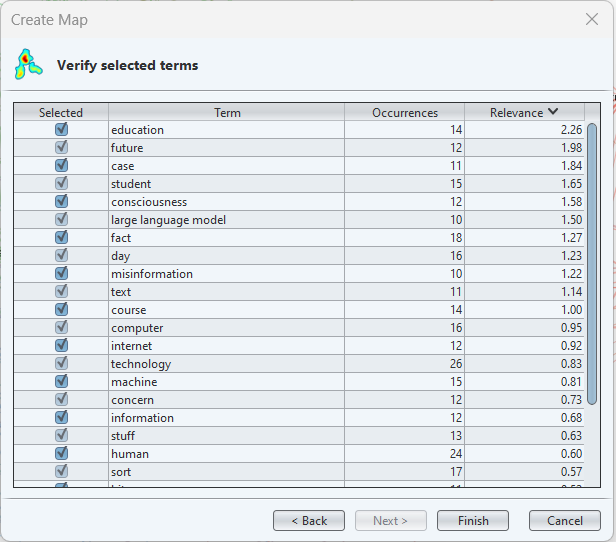


1. Verify selected terms: This stage allow user the sort the terms based on their relevance to the analysis. Irrelevant terms can deleted for further analysis.
